# Pentacyclic Triterpene Distribution in Various Plants – Rich Sources for a New Group of Multi-Potent Plant Extracts

**DOI:** 10.3390/molecules14062016

**Published:** 2009-06-04

**Authors:** Sebastian Jäger, Holger Trojan, Thomas Kopp, Melanie N. Laszczyk, Armin Scheffler

**Affiliations:** 1 Carl Gustav Carus-Institute, Am Eichhof 30, D-75223 Niefern-Öschelbronn, Germany; 2 Betulin-Institute, Blumenstrasse 24, D-64297 Darmstadt, Germany; E-Mail: m.laszczyk@betulin-institut.de (M-N.L.)

**Keywords:** lupane, oleanane, ursane, triterpene dry extract, active plant extracts, triterpene distribution

## Abstract

Pentacyclic triterpenes are secondary plant metabolites widespread in fruit peel, leaves and stem bark. In particular the lupane-, oleanane-, and ursane triterpenes display various pharmacological effects while being devoid of prominent toxicity. Therefore, these triterpenes are promising leading compounds for the development of new multi-targeting bioactive agents. Screening of 39 plant materials identified triterpene rich (> 0.1% dry matter) plant parts. Plant materials with high triterpene concentrations were then used to obtain dry extracts by accelerated solvent extraction resulting in a triterpene content of 50 ‑ 90%. Depending on the plant material, betulin (birch bark), betulinic acid (plane bark), oleanolic acid (olive leaves, olive pomace, mistletoe sprouts, clove flowers), ursolic acid (apple pomace) or an equal mixture of the three triterpene acids (rosemary leaves) are the main components of these dry extracts. They are quantitatively characterised plant extracts supplying a high concentration of actives and therefore can be used for development of phytopharmaceutical formulations.

## Introduction

**Figure 1:**
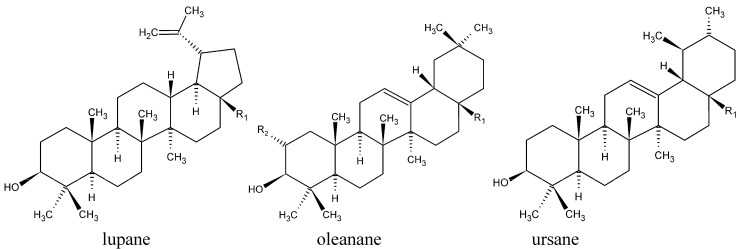
Molecule structures of lupane-, oleanane- and ursane triterpenes investigated here.

Consumption of fruit and vegetables has been associated with a lower incidence of cancer and other diseases. Diets, especially along the Mediterranean coast, are correlated with healthiness [1]. Mediterranean spices and fruits contain, besides other nutraceuticals, pentacyclic triterpenes from the lupane, oleanane and ursane groups (see Figure 1 and Table 1), that are regularly isolated as active substances from these plants. For example, they can be found in rosemary and other spices of the *Lamiaceae* family as well as within olive leaves and fruit. Virgin olive oil contains up to 197 mg/kg triterpenes, indicating the importance of these substances as nutraceuticals [1,2,3,4]. Furthermore, the bio-guided fractionation of several hundred plant extracts led to the isolation of betulinic acid (BA), oleanolic acid (OA) and ursolic acid (UA) as the active principles [5]. Apples are among the fruit most consumed worldwide and anti-tumoral effects from apples are correlated with the fruit peel [6] which contains OA, UA and maslinic acid (MA) [7]. Known sources for triterpenes are mainly plant surfaces such as stem bark or leaf and fruit waxes [8].

**Table 1:** Triterpene characterisation.

Triterpene family	Triterpene	R_1_	R_2_	M [g/mol]	Abbreviation
lupane	lupeol	CH_3_		426.70	LU
	betulin	CH_2_OH		442.72	BE
	betulinic acid	COOH		456.71	BA
oleanane	β-amyrin	CH_3_	H	426.70	bAM
	erythrodiol	CH_2_OH	H	442.72	ER
	oleanolic acid	COOH	H	456.71	OA
	maslinic acid	COOH	OH	472.70	MA
ursane	α-amyrin	CH_3_		426.70	aAM
	uvaol	CH_2_OH		442.72	UV
	ursolic acid	COOH		456.71	UA

The pharmacological relevance of these triterpenes has increased during the last two decades demonstrating multi-target properties such as wound healing, anti-inflammatory, anti-bacterial, anti-viral, hepatoprotective and anti-tumoral effects, combined with low toxicity [9,10,11,12,13]. Therefore triterpene plants are a source of actives for phytopharmaceutical development. Knowledge of the occurrence of triterpenes in plants is extensive [8,14] but little is known about their quantitative distribution. The aim was to search for rich sources of triterpenes from the lupane, oleanane and ursane group (see Figure 1 and Table 1) as materials for triterpene extraction. Recently, a new kind of one-step plant extract with 78% betulin (BE) was prepared from birch bark [15]. Thus we investigated whether this kind of extraction procedure could be adapted to other triterpene plants in order to gain highly concentrated extracts with triterpene leading substances other than BE.

Various galenic possibilities are known for the preparation of triterpenes. As ingredients of medicinal plants, triterpenes are used in traditional herbal medicine [16]. A self-nanoemulsified drug delivery system exists for oral delivery of OA [16]. The preparation of a semi-solid topical formulation of triterpenes is realised for instance with the above mentioned triterpene dry extract from the outer bark of birch. It has been used successfully in treating actinic keratoses [17]. Parenteral applications of triterpenes could be achieved by liposomal encapsulation [18] or complexation with cyclodextrins [19]. Therefore the galenic possibilities of triterpene rich plant extracts are wide ranging. Here we present the preparation of lupane, oleanane, and ursane extracts.

## Results and Discussion

### Triterpene distribution within various plant materials

**Figure 2:**
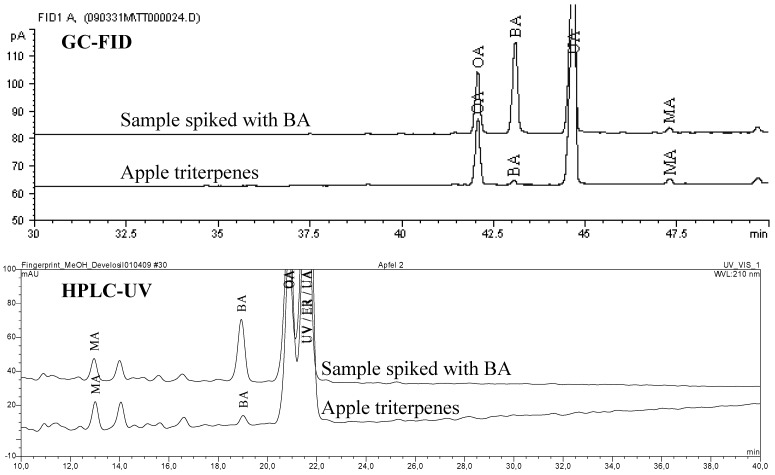
Confirmation of BA in apples using standard addition and GC-FID / HPLC-UV.

Thirty nine known triterpene plants were quantified for their triterpene content (GC-FID) with a limit of detection (LOD) and a limit of quantification (LOQ) within the dry matter (dm) of 0.03 g/100 g (%) and 0.10%, respectively. The measured amounts, listed in Table 2, show the dominance of BE in birch bark [15]. However, the triterpene acids BA, OA and UA are frequent constituents of various plants, reaching concentrations up to 2**-**3% (BA in plane bark, OA in olive leaves and UA in rosemary leaves). For some detected triterpenes there were no literature references available. That is why we obtained preliminary evidence of identity by spiking concentrated extracts with standards. The chromatographic separation was performed using GC-FID and HPLC-UV respectively and the chromatograms were inspected visually for peak purity of spiked triterpenes. A methanol gradient was developed on a C-30 column to nearly separate all tested triterpenes by HPLC as a complementary method to GC. The following example illustrates the determination of BA within apple pomace (see Figure 2). This confirmation technique does not unequivocally identify the marked (^+^) substances within Table 2 but it gives a strong hint to the peak identity. Because pentacyclic triterpenes display a large variety of similar molecular skeletons, the unknown substances may well be different to the identified molecules or they may be co-eluting with similar substances.

The quantification method applied by Silva *et al.* uses Soxhlet extraction with methanol for three hours [20]. Razborsek *et al*. combines solvent extraction with solid phase and size exclusion extraction prior to GC-MS of silylated triterpenes [21]. The accelerated solvent extraction (ASE) method presented here reaches complete extraction within 45 min without any further clean-up step prior to GC-FID of silylated triterpenes [15,22]. Using hydrogen as the mobile phase and a more polar ZB-35 column, the separation of triterpenes is better than with helium on a HP-5 column [21]. In any case, MS detection is superior to FID in terms of sensitivity and peak characterisation. The HPLC-method presented by Sanchez-Avila *et al*. separates the triterpene acids and dialcohols using acetonitrile as mobile phase [23]. The HPLC method used here for identification is not able to separate UV, ER and UA but it additionally shows the triterpene mono alcohols. Furthermore acetonitrile was not used as the mobile phase. This has become cost-effective since the acetonitrile crisis of 2008/2009 [24]. 

**Figure 3:**
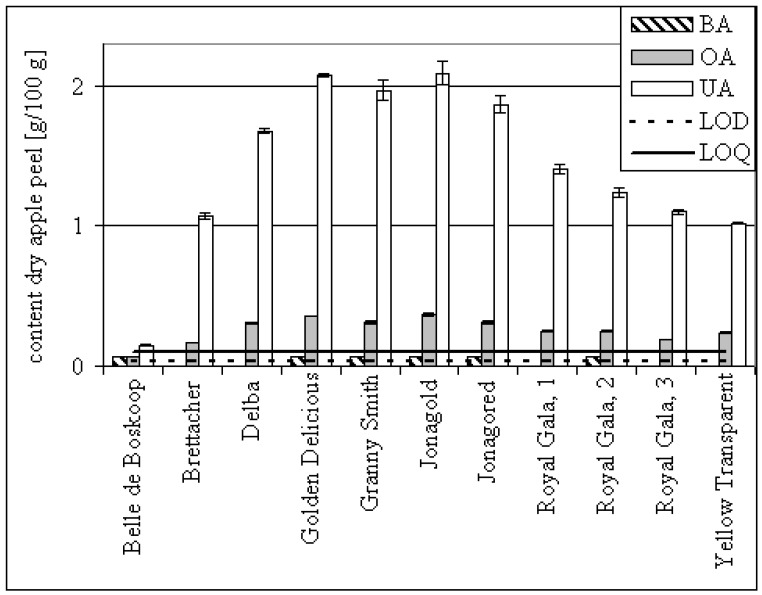
Triterpene acid content of dried apple peels. Error bars: ± standard deviation of analysis. Detectable triterpenes (< LOQ) are set to 0.07% arbitrarily.

The following discussion compares published amounts with our results. Duke lists the UA amount of bearberry leaves as 0.4 – 0.75%. Possibly it was fresh plant material so the 1.24% we quantified in the dry matter seemed comprehensible [14]. The OA concentration of pot marigold flowers was reported to be 2%, but our investigations showed < 0.1% [25]. Eleven batches of dried apple peel were analysed for their triterpene content. On average, they contained detectable amounts of BA, OA 0.28% (confidence interval 0.04%, α = 0.05) and UA 1.43% (confidence interval 0.35%, α = 0.05) (see Table 2). Dried peel of the Cox’s Holsteiner variety was reported to contain 0.3% UA and Fuji 0.7% UA [6,26]. These varieties were not analysed in our study, but the amounts we found seem to be reasonable, since our data for dried peel are within the range of 0.2 and 2.1% UA (see Figure 3). The differences in amounts appear to correlate with the quantity of wax distinguishable on the surface of the apple. The measured amounts for normal sized (65 – 80 mm) apples are about two decimal orders of magnitude lower than those published by Frighetto *et al*., who found ~10 mg UA on one Gala apple [27]. As an example, the OA and UA amounts on the surfaces of individual apples were calculated for the varieties Jonagold, Jonagored and Royal Gala (see Table 3).

**Table 2:** Triterpene distribution within various dry plant materials.

Binomial name	Common name	Plant part	[g/100 g dry matter]									ID
			LU	BE	BA	bAM	ER	OA	aAM	UV	UA	
*Aesculus hippocastanum*	horse-chestnut	leaves				det						[8]
*Aloe vera*	aloe vera	leaves	0.10									[28]
*Arctostaphylos uva-ursi*	bearberry	leaves	0.29		0.12	0.10	0.18	0.27	0.25	0.35	1.24 (0.40–0.75)	[8, 14]
*Betula alba*	birch	bark	(0.9–2.1)	(10.5–18.3)	(0.5–1.3)		(0.2–0.4)	(0.1–1.1)				[15]
*Calendula officinalis,*	pot marigold	flowers	det					det (2.0)				[25, 29]
*Centaurium erythraea*	common centaury	herb				det		0.16	det			[8]
*Coffea arabica*	coffee	leaves						det			1.80	[30]
*Cornus mas*	european cornel	leaves									0.15	[31]
*Crataegus*	hawthorn	leaves, flowers						0.10			0.52	[32]
*Eucalyptus*	euca-lyptus	leaves			0.84			0.31			1.17	[33]
*Lavandula angustifolia*	lavender	leaves			0.13			0.45			1.59	[8]
*Lavandula angustifolia*	lavender	flowers			0.12			0.40			1.05	[8]
*Malus domestica*	11 diff. apples	fruit peel			det^+^			0.28 (0.07)			1.43 (0.3–0.7)	[6, 26]
*Malus domestica*	apple pomace	pomace						0.16			0.80	[26]
*Melissa officinalis*	lemon balm	*leaves*						0.16			0.67	[34]
*Nerium oleander*	oleander	leaves			0.11			0.37			1.27	[35]
*Ocimum basilicum*	sweet basil	leaves						det			det (0.3)	[20, 36]
*Olea europeae*	olive	leaves					det (0.3)	3.10 (1.3)		(0.3)	0.18 (0.5)	[23]
*Olea europeae*	olive	bark		det^+^	det^+^		det^+^	0.98^+^			det^+^	
*Olea europeae*	olive	fruit^*^						0.21 (0.09–0.16)				[37]
*Olea europeae*	olive	pomace						0.18				[38]
*Origanum majorana*	marjoram	leaves						0.19			0.66	[39]
*Origanum vulgare*	oregano	leaves						det			0.28	[8]
*Pimpinella anisum*	anisseed	seed				det						[8]
*Plantago major*	greater plantain	*leaves*						det			0.21	[40]
*Platanus acerifolia*	planes	bark		det^+^	2.44 (3.3)			det^+^				[41]
*Prunus cerasus*	sour cherry	unripe fruit									det	[8]
*Pyrus communis*	pear williams'	fruit peel									0.20	[42]
*Rosmarinus officinalis*	rosemary	leaves			1.53 (0.61)			1.23 (0.91)			2.95 (1.58)	[21]
*Salvia officinalis*	sage	leaves			(0.02)	det		0.67 (0.76)			1.80 (1.52)	[21]
*Sambucus nigra*	black elder	leaves						0.12			0.58	[8, 43]
*Sambucus nigra*	black elder	*bark*		det	det			0.08			0.32	[8, 43]
*Satureja montana*	winter savory	leaves			(0.04)			0.14 (0.54)			0.49 (0.09)	[21]
*Solanum lycopersicum*	tomato	fruit peel				det						[44]
*Syringa*	lilac	*leaves*									det	[8]
*Syzygium aromaticum*	clove	flower			det^+^			1.65			det^+^	[45]
*Thymus vulgaris*	common thyme	leaves						0.37			0.94	[46]
*Verbena officinalis*	common vervain	herb						det			0.17	[47]
*Viscum album*	mistletoe	sprouts			det (0.05)			0.86 (0.16)				[22]
*Vitis vinifera*	grape vine	leaves	det									[8]

Det = detectable (> LOD, < LOQ) ; ID = reference for identity and amount of triterpene(s) within that plant ; ^+^ identity was confirmed by GC-FID and HPLC-UV using standard addition ; ^*^ unripe, green fruit without endocarp ; ( ) = figures in brackets are published amounts (for citation see the column “ID” of Table 2).

**Table 3:** Triterpene distribution within apples.

Apple	OA per apple [mg]	UA per apple [mg]
Jonagold	0.018	0.100
Jonagored	0.010	0.058
Royal Gala 1	0.009	0.052
Royal Gala 2	0.008	0.038

Sanchez-Avila *et al*. determined a maximum concentration of 1.3% OA, 0.3% ER, 0.3% UV, 0.5% UA and 0.3% MA in fresh olive leaves [23]. Because dried plant material was analysed in our study, the results are consistent with published data. Olive tree bark was analysed for the first time for its triterpene composition, showing mainly OA. Olives contain low amounts of bAM, aAM, ER, UV, 3-*epi*-betulin and higher amounts of OA and MA [37]. Stiti *et al.* quantified the triterpene concentration during the ripening of olives and found an rise and fall in the OA and MA concentrations (OA: 0.09 - 0.16%; MA 0.08 – 0.23% respectively within the dry matter) during weeks 12 and 30 after flowering [37]. The OA concentration measured here in green olives without endocarp was 0.2%, which correlates with the amounts measured within the whole fruit by Stiti *et al*. Olive pomace from virgin oil extraction contained 0.18% OA and 0.37% MA respectively in the dry matter. Because triterpene acids dissolve poorly during cold extraction (maximum 179 mg/kg [1]), the amounts measured within the pomace are to be expected. 

The quantitative results of BA in plane bark, where Galgon *et al.* found 3.3% [41], in comparison to our 2.4% are approaching each other. The Lamiaceae family is an especially good source for UA, OA and BA, reaching the highest concentration measured within rosemary leaves. However, OA has been found in *Lavendula latifolia* L., but not yet in *angustifolia* L. [8]. The UA concentration within sweet basil was < 0.1% whereas 0.3% was found by HPLC-UV [20]. This difference could be due to seasonal variations. Eiznhamer lists the BA concentration of rosemary leaves as 8.6%, but this is not mentioned in the article he cited [2,48]. Razbrorsek *et al*. found 0.6% BA, 0.9% OA and 1.6% UA in rosemary leaves (per dry weight) [21]. The concentrations we measured are about twice as much as found by Razbrosek *et al*., who also quantified the triterpene acids in *Salvia officinalis* L. and found them present in similar amounts as we did. In *Satureja montana* L. Razbrosek *et al*. found more OA than UA but the same summed amount as we quantified [21]. The triterpene content of mistletoe previously reported by our group was indicated as the amount in the fresh plant material explaining the different amount measured in another dried plant material here [22]. Scher *et al*. demonstrated that the OA content decreases during maturation of mistletoe leaves (1.2 – 0.8%) but no difference of the triterpene contend was found between the subspecies *album*, *abietis* and *austriacum* [49]. 

### Preparation of triterpene dry extracts (TE)

Apart from LU and BE in birch bark [15], triterpene alcohols did not reach concentrations above 1% in the plant materials tested. Therefore preparative extractions were made only for triterpene acids. We previously reported an extraction method with heated *n*-heptane, were a triterpene dry extract (TE) of birch bark is formed [13,15]. This method was used for the extraction of triterpene rich and highly available plant materials. Birch bark, apple pomace and olive pomace are waste materials accumulated in large amounts in the timber, pectin and oil industries. 

**Figure 4:**
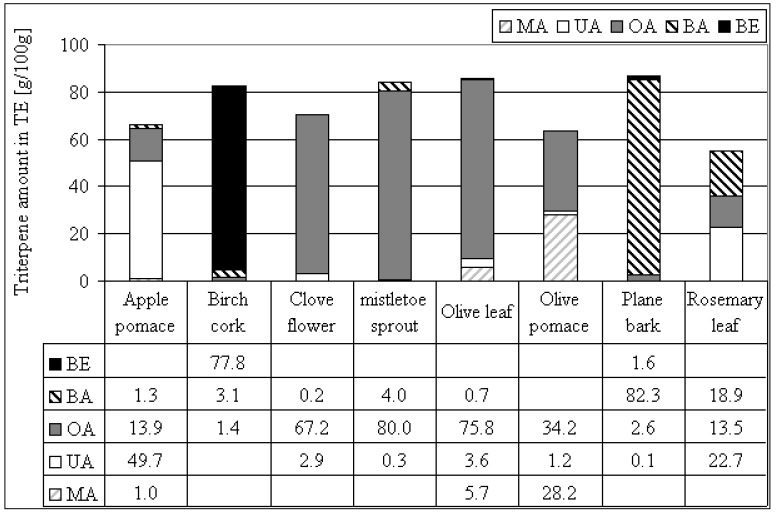
Triterpene amount within triterpene dry extracts (TE) from various plant materials.

Using different plant materials, it is possible to prepare triterpene dry extracts with a triterpene content of more than 50% and a dominance of BE (birch) [15], BA (plane), OA (olive, mistletoe, clove) or UA (apple) (see Figure 4). More or less equal proportions of BA, OA and UA are obtained by the extraction of rosemary leaves. Using olive pomace instead of olive leaves leads to higher proportions of MA, a reduction of OA and a reduction of the total triterpene content. In comparison to our method Guinda reported an olive leaf extract containing 93% OA after organic extraction and crystallisation, which is 17% more than we obtained [50]. The extract of olives tested by Juan *et al.* was prepared by chloroform extraction resulting in 0.1% OA and 0.04% MA with traces of ER [3]. The extraction method presented here for olive leaves yielded 75.8% OA and a total of 85.9% triterpenes (OA, BA, UA and MA). The recovery rates of triterpenes from the plant material into TE were calculated for apple pomace (31%), mistletoe sprouts (65%), olive leafs (32%), olive pomace (34%) and rosemary leaves (40%). Triterpenes could be lost due to incomplete extraction or due to incomplete precipitation in *n*-heptane after extraction.

These triterpene dry extracts represent a new group of primary plant extracts because of their high amount of actives and the grade of identification. Hitherto, extracts of *Ginkgo biloba* have been rated as highly characterised, where 30% is chemically well characterised and the 6 classes of substances make up to 70% of the extract [51].

## Conclusions

The determination of lupane, oleanane and ursane triterpenes led to the identification of several triterpene rich plant materials. Apart from dialcohol triterpenes within birch bark, triterpene acids were the only substances tested, reaching concentrations above 1% in the dry plant material. Some of the triterpene rich plant materials are common foodstuffs consumed in large amounts in Mediterranean countries. Therefore the correlation of a triterpene acid rich nutrition and the beneficial effects of consuming Mediterranean food should be investigated in more detail. The preparative extraction of selected plant materials with heated *n*-heptane leads to primary plant extracts containing concentrations of triterpene acids of > 50% showing that this technique is suitable for the preparation of extracts of materials other than birch bark. The triterpene acid distribution within the plant extract depends on the plant material, which is why extracts with mainly BA, OA or/and UA can be prepared. These triterpene acids are known as potent actives and, within these highly characterised plant extracts, form an ideal starting material for pharmaceutical development. 

## Experimental

### General

Various plant materials were quantified by accelerated solvent extraction (ASE) / GC-FID for their quantitative triterpene distribution. This method uses external calibration with standards and was validated for birch bark and mistletoe sprouts as examples according to ICH guidelines. Triterpene dry extracts were prepared from some appropriate plant parts by ASE and characterized by the GC-method described above. A HPLC-UV method was developed to supplement confirmation of some triterpenes not yet described for a particular species.

**Table 4:** Plant material and plant parts used for analysis and extraction of TE.

Binomial name	Common name	Plant part	Plant origin, identification; batch or collection
*Aesculus hippocastanum*	horse-chestnut	leaves	collection *, D-75223 Niefern; collection: 06.08.07
*Aloe vera*	aloe vera	leaf	greenhouse *, D-75223 Niefern; collection: 06.08.07
*Arctostaphylos uva-ursi*	bearberry	leaves	Linden Apotheke, D-75223 Niefern; batch: 22.06.07
*Calendula officinalis,*	pot marigold	flowers	Linden Apotheke, D-75223 Niefern; batch: 05/2009
*Centaurium erythraea*	common centaury	herb	Linden Apotheke, D-75223 Niefern; batch: 22.06.07
*Coffea*	coffee	leaves	greenhouse*, D-75223 Niefern; collection: 13.07.07
*Cornus mas*	european cornel	leaves	collection *, D-75223 Niefern; collection: 06.08.07
*Crataegus*	hawthorn	leaves & flowers	Linden Apotheke, D-75223 Niefern; batch: 22.06.07
*Eucalyptus*	eucalyptus	leaves	Heinrich Klenk, D-97525 Schwebheim; batch: 2011 A 060411 01
*Lavandula angustifolia*	lavender	leaves	collection *, D-75223 Niefern; collection: 06.08.07
*Lavandula angustifolia*	lavender	flowers	collection *, D-75177 Pforzheim; collection: June 08
*Malus domestica*	apple	peel	brettacher, collection *, D-72805 Lichtenstein; collection: autumn 06
*M. domestica*	apple	peel	jonagored, E. Grundler, D-78333 Espasingen; batch: L 20 111
*M. domestica*	apple	peel	jonagold, Salemfrucht, D-88682 Salem; batch: L 24218
*M. domestica*	apple	peel	granny smith, Plus Warenhaus, 45466 Mühlheim; batch: August 07
*M. domestica*	apple	peel	golden delicious, Plus Warenhaus, 45466 Mühlheim; batch: August 07
*M. domestica*	apple	peel	royal gala 1, OGM Richard Schsugg, D-88097 Eriskirch-Wolfzennen; batch: 17.08.07
*M. domestica*	apple	peel	royal gala 2, Clementi Gabr. GmbH Leifers-Südtirol; batch: 17.08.07
*M. domestica*	apple	peel	royal gala 3, Südtiroler Apfel g.g.A. VOG Gen.landw.Ges. I – 39018 Terlan, batch: 17.08.07
*M. domestica*	apple	peel	belle de boskoop, collection *, D-76534 Geroldsau; collection: 30.09.08
*M. domestica*	apple	peel	yellow transparent, collection *, D-75223 Niefern; collection: October 08
*M. domestica*	apple	peel	delba, collection *, D-75203 Königsbach; collection*: July 07
*M. domestica*	apple	pomace	Herbstreith & Fox, D-75305 Neuenbürg; Herbavital F12 batch: 14.08.07
*Melissa officinalis*	lemon balm	leaves	collection *, D-75223 Niefern; collection: 06.08.07
*Nerium oleander*	oleander	leaves	collection *, D-75446 Wiernsheim; collection: 19.04.07
*Ocimum basilicum*	sweet basil	leaves	Zielpunkt Warenhandel, Plus Warenhaus, 45466 Mühlheim; batch: MHD 2009
*Olea europeae*	olive	leaves	collection **, Greece; collection: 01.07.07
*Olea europeae*	olive	bark	collection **, Greece; collection: 01.07.07
*Olea europeae*	olive	fruit	without endocarp, Las Cuarenta, Plus Warenhaus, D-45466 Mühlheim; batch: L-05/02/2010
*Olea europeae*	olive	pomace	Merum Verlag, I-51035 Lamporecchio; batch: 12/08
*Origanum majorana*	marjoram	leaves	Zielpunkt Warenhandel, Plus Warenhaus, 45466 Mühlheim; batch: MHD 2009
*Origanum vulgare*	oregano	leaves	Ostmann Gewürze, D-33596 Bielefeld; batch: L7026CD
*Pimpinella anisum*	aniseed	seed	Ostmann Gewürze, D-33596 Bielefeld; batch: L6280AS
*Plantago major*	greater plantain	leaves	collection *, D-75223 Niefern; collection: 06.08.07
*Platanus*	planes	bark	collection *, D-75223 Niefern; collection: 08.03.07
*Prunus cerasus*	sour cherry	fruit	collection *, D-75223 Niefern; collection: 15.05.07
*Pyrus communis*	pear williams'	peel	Fruit du monde, Plus Warenhaus, 45466 Mühlheim; batch: L 11/5
*Quercus*	oak tree	leaves	collection *, D-75223 Niefern; collection: 05.06.07
*Rosmarinus officinalis*	rosemary	leaves	Heinrich Klenk, D-97525 Schwebheim; batch: 2261 A 051201 03
*Salvia officinalis*	sage	leaves	Ostmann Gewürze, D-33596 Bielefeld; batch: 6123AA
*Sambucus nigra*	black elder	leaves	collection *, D-75223 Niefern; collection: 06.08.07
*Sambucus nigra*	black elder	bark	collection *, D-75223 Niefern; collection: 06.08.07
*Satureja montana*	winter savory	leaves	collection *, D-75223 Niefern; collection: 06.08.07
*Solanum lycopersicum*	tomato	peel	Rewe, D-75223 Niefern; batch: 26.03.07
*Syringa*	lilac	leaves	collection *, D-75223 Niefern; collection: 13.05.07
*Syzygium aromaticum*	clove	flower	Ostmann Gewürze, D-33596 Bielefeld; batch: L6326DB
*Thymus vulgaris*	common thyme	leaves	Ostmann Gewürze, D-33596 Bielefeld; batch: L6300DD
*Verbena officinalis*	common vervain	herb	Linden Apotheke, D-75223 Niefern; batch: 22.06.07
*Viscum album ssp. album*	mistletoe (apple tree)	sprouts	collection *, D-75223 Niefern; collection: 12.07.07
*Vitis vinifera*	grape vine	leaves	collection *, D-75223 Niefern; collection: 06.08.07

^*^ wild collection identified by A. Heinze, S. Jäger, ** M. Kikidaki, Carl Gustav Carus-Institute.

### Plant material

Plant material was collected or purchased from various sources and identified as described in Table 4. A voucher specimen of each batch was deposited in the archive of Carl Gustav Carus-Institute, Niefern-Öschelbronn, Germany.

### Quantification of triterpenes within plant material

**Figure 5:**
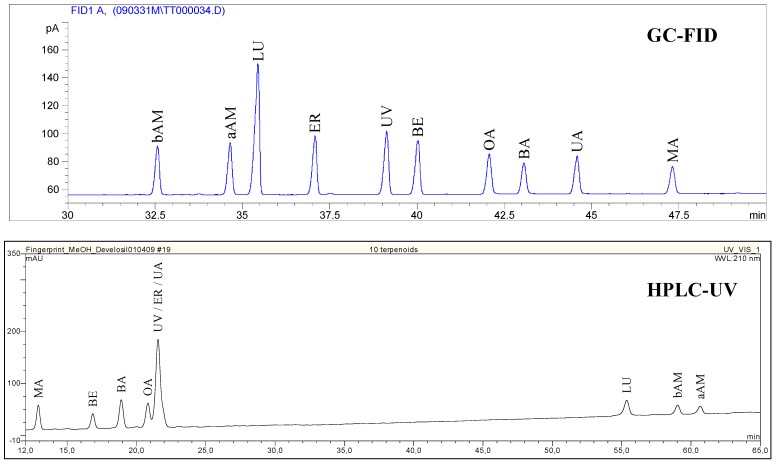
GC-FID and HPLC-UV chromatogram of triterpene standards.

Apples were peeled with an apple peeler resulting in 9 – 11% apple peel as reported by He and Liu [7]. All fresh plant parts were dried at 80°C (± 5°C) for 3 h, and all dried plant materials (3 g per analysis) were extracted using accelerated solvent extraction (ASE) with ethyl acetate at 1,450 psi and 120°C [15,22]. Quantification of silylated triterpenes within the extract was performed by GC-FID with external standard calibration (see Table 5 and Figure 5). This extraction and quantification method was validated and described for birch bark and mistletoe sprouts [15,22]. Each sample was analysed in triplicate (or duplicate) resulting in a coefficient of variation < 5%. According to validations the limit of detection (LOD) was 0.03 g/100g and the limit of quantification 0.10 g/100g dried plant material.

**Table 5:** Triterpene standards.

Standard	Batch	Manufacturer
lupeol (LU)	061K1772	Sigma-Aldrich, Munich, Germany
betulin (BE)	BE 150307	Carl Gustav Carus-Institute, Niefern, Germany
betulinic acid (BA)	34255520	Carl Roth, Karlsruhe, Germany
β-amyrin (bAM)	0016 S 16	Extrasynthese, Genay Cedex, France
erythrodiol (ER)	114611124804002	Fluka, Sigma-Aldrich, Munich, Germany
oleanolic acid (OA)	38681978	Carl Roth, Karlsruhe, Germany
α-amyrin (aAM)	0015 S 06	Extrasynthese, Genay Cedex, France
ursolic acid (UA)	2208J	MP Biomedicals, Ilkirch, France
maslinic acid (MA)	184071-200621	Cayman Chemicals Ann Arbor, Michigan USA

### Preparation and characterization of triterpene dry extracts (TE)

For the preparation of TE of plant materials, the dried plant parts were extracted by ASE with *n*-heptane at 1450 psi and 120°C as described for birch bark [13]. The dried precipitate was analysed by GC-FID [15]. 

### Confirmation of triterpene identity

Confirmation of triterpene identity was performed using GC-FID and HPLC-UV by standard addition, if it was an unknown triterpene for that plant. Triterpenes are concentrated within the TE. For this reason these extracts were used for the identity confirmation. GC-analysis was performed using the method described above. For HPLC-UV analysis, a saturated sample solution was prepared in methanol/water 80/20 (v/v) and filtered (0.45 µm, Millex-HV, Millipore). 100 µL were injected on a Develosil RP aqueous Column (5 µm, 250 x 4.6 mm, Phenomenex). The flow rate was 1.5 mL/min with a gradient starting at methanol/water 80/20 (v/v; containing 0.1% trifluoracetic acid (TFA)), increasing the methanol (+ 0.1% TFA) concentration to 100% within 60 min and keeping that concentration constant for 10 min. The detection wavelength was 210 nm and the standards described in Table 5 were used for peak identification (see Figure 5 for a standard chromatogram). Chromatograms were inspected visually for peak symmetry or shoulders of spiked triterpenes.

### Statistics

Microsoft Excel was used for the calculation of average, standard deviation and the confidence interval (CI; α = 0.05). 
